# Neuroimmune Interactions in Pancreatic Cancer

**DOI:** 10.3390/biomedicines13030609

**Published:** 2025-03-02

**Authors:** Jun Cheng, Rui Wang, Yonghua Chen

**Affiliations:** 1 Operating Room, Department of Anesthesiology, West China Hospital/West China School of Nursing, Sichuan University, Chengdu 610041, China; ella20210305@163.com; 2 Division of Pancreatic Surgery, Department of General Surgery, West China Hospital, Sichuan University, Chengdu 610041, China; wr1293634291@163.com; 3 Department of General Surgery, West China Tianfu Hospital of Sichuan University, Chengdu 610041, China

**Keywords:** pancreatic cancer, neuroimmune, peripheral nervous system, immune system, neuroimmune interactions

## Abstract

Pancreatic ductal adenocarcinoma (PDAC) is a highly aggressive primary malignancy, and recent technological advances in surgery have opened up more possibilities for surgical treatment. Emerging evidence highlights the critical roles of diverse immune and neural components in driving the aggressive behavior of PDAC. Recent studies have demonstrated that neural invasion, neural plasticity, and altered autonomic innervation contribute to pancreatic neuropathy in PDAC patients, while also elucidating the functional architecture of nerves innervating pancreatic draining lymph nodes. Research into the pathogenesis and therapeutic strategies for PDAC, particularly from the perspective of neuroimmune network interactions, represents a cutting-edge area of investigation. This review focuses on neuroimmune interactions, emphasizing the current understanding and future challenges in deciphering the reciprocal relationship between the nervous and immune systems in PDAC. Despite significant progress, key challenges remain, including the precise molecular mechanisms underlying neuroimmune crosstalk, the functional heterogeneity of neural and immune cell populations, and the development of targeted therapies that exploit these interactions. Understanding the molecular events governing pancreatic neuroimmune signaling axes will not only advance our knowledge of PDAC pathophysiology but also provide novel therapeutic targets. Translational efforts to bridge these findings into clinical applications, such as immunomodulatory therapies and neural-targeted interventions, hold promise for improving patient outcomes. This review underscores the need for further research to address unresolved questions and translate these insights into effective therapeutic strategies for PDAC.

## 1. Introduction

The nervous system and immune system communicate throughout the body and in the central nervous system, which can recognize danger simultaneously and respond in both a simultaneous and coordinated fashion to regulate tissue and organ function in homeostasis and diseases [1,2,3,4,5]. However, the field of neuroimmunology has undergone a recent renaissance, and evidence suggests that nervous system and immune system communication is far more complex than previously realized.

Neuroimmunology studies to date have tended to focus on interactions between nerves and immune systems at the local level. Peripheral neuroimmune interactions critically regulate systemic and local tissue immunity. Novel data show that the immune system and nervous system play mutual roles in the body and are essential in immune, metabolism-related, neoplastic, and other diseases [6,7,8].

With its neuroimmunological properties, the pancreas is richly supplied with neuronal connections that mediate crucial physiological activities. In addition, pancreatic draining lymph nodes have pancreatic innervation and exert some immunomodulatory effects [9]. Notably, research on the pathogenesis and therapeutic options for treating pancreatic ductal adenocarcinoma (PDAC), including recently identified local forms, from the viewpoint of crosstalk of the neuroimmune network and intervention techniques is at the cutting edge. Current evidence reveals the neuroimmune crosstalk occurring in PDAC, explaining the role of interactions between different types of nerve and immune cells in PDAC in terms of tumor formation, development, and metastasis [10,11,12]. Importantly, these neuroimmune interactions have significant clinical implications. Studies have shown that neural invasion and neuroimmune signaling contribute to the immunosuppressive tumor microenvironment, which may explain the poor prognosis and limited therapeutic efficacy observed in PDAC patients. These findings underscore the potential of neuroimmune research to translate into novel therapeutic approaches that address the unmet clinical needs in PDAC.

In this review, we focus specifically on neuroimmune interactions associated with PDAC, aiming to elucidate the value of neuroimmune crosstalk in PDAC pathogenesis and intervention through the effects of different types of neural and immune cells on PDAC. A systematic literature search was conducted using the following electronic databases: PubMed and Web of Science. The search terms included combinations of the following keywords: “pancreatic ductal adenocarcinoma”, “neuroimmune interactions”, “neural invasion”, “autonomic nervous system”, “tumor microenvironment”, and “immune modulation”.

## 2. Neuroanatomy in the Pancreas and Draining Lymph Nodes

The anatomy of pancreatic innervation includes sympathetic efferent, parasympathetic efferent, vagal afferent, spinal afferent, and enteropancreatic innervation [13]. In the human pancreas, these different types of nerves have distinct innervation characteristics. Using three-dimensional imaging, Chien and colleagues revealed that nerve fibers positive for substance P as the afferent nerve are located at the base of the interlobular duct and that nerve fibers positive for vesicular acetylcholine transporter and tyrosine hydroxylase as the efferent nerve exist in the periacinar and perivascular spaces, which reach the islet along a blood vessel [14]. Neurons and glial networks enter the islet core, and sympathetic and parasympathetic nerves reside in the immediate microenvironment [15]. However, few parasympathetic cholinergic axons innervate the islets in humans, and the sympathetic nerves entering the islets preferentially innervate the vascular smooth muscle cells in islets [16]. The sympathetic nervous system constricts blood vessels in many parts of the body to ensure the stability of the circulatory system under special circumstances [16]. Nerves not only extend to pancreatic parenchyma cells but also some mesenchymal cells. However, further studies are needed to confirm the existence and function of unique connections between nerve cells and various mesenchymal cells.

More recently, novel findings on the functional structure of nerves innervating pancreatic draining lymph nodes have been reported in terms of neuroimmune regulation [9]. Lymph nodes serve as immune organs to filter lymphatic fluid and initiate local adaptive immune responses. The peripheral nervous system communicates specifically with the immune system through local interactions that provide the structural basis for complex immune and neural response networks [17]. By identifying a molecularly distinct and heterogeneous population of sensory neurons with the capacity to affect lymph node function and homeostasis, Huang et al. recently established lymph nodes as a point of convergence between the sensory nervous system and the immune system [18].

Regarding experimental animals, the presence of norepinephrine innervation in the popliteal and mesenteric lymph nodes of mice has long been detected by fluorescein histochemistry [19]. Sympathetic nerves, including perivascular and discrete structures, are observed in human inguinal lymph nodes, and the number of sympathetic nerves varies between compartments and between and within individuals [20]. According to a study of human pancreas specimens, sympathetic innervation of secondary lymphoid organs (such as lymph nodes) varies by species and is influenced by certain physiological or pathological conditions [21]. By placing a suction electrode and recording a field action potential with microelectrodes, projection of pancreatic nerves to mouse lymph nodes was demonstrated by stimulation of pancreatic nerves [9].

## 3. Neural Plasticity in PDAC

The neural plasticity of PDAC is evidenced by concurrent neuronal activation in peripheral nerves, the spinal cord, and higher central nervous system regions [22]. Sympathetic nerve innervation of the pancreas is significantly lower in patients with PDAC than in patients with a healthy pancreas, and the number of sympathetic fibers in PDAC patients without nerve invasion is markedly higher than in those with nerve invasion [23]. In contrast, there is no significant change in parasympathetic or cholinergic nerve distribution [23].

In terms of spatial variation in the pancreatic nerve distribution, increased nerve density and hypertrophy compared with those in the normal pancreas are typical features of PDAC [24]. The density of intrapancreatic nerves in patients with PDAC tends to decrease toward the center of the tumor [2]. In mice, nerve hypertrophy and infiltration gradually follow the progression of PDAC, and hypertrophy of the nerve is associated with fibrosis of the corresponding area and atrophy of pancreatic acinar cells [25]. The mean percentage of sympathetic nerve fibers per nerve in the pancreas of PDAC patients is significantly lower than that in the pancreas of healthy patients [23], which may be partly explained by a decrease in the number or volume of sympathetic nerves or both. In patients with PDAC, increased nerve size is associated with decreased numbers of cholinergic and adrenergic nerve fibers and enhanced neural nesting immunoreactivity [23]. Indeed, there is a correlation between the volume and number of pancreatic nerves. In addition to the neurological changes described above, changes in neurotrophic factor expression occur prior to tumor formation, and the nervous system is involved in all PDAC processes, including the period before cancer development [25].

## 4. Systemic Influences of Neuroimmune Signaling

While local neuroimmune interactions within the tumor microenvironment have been increasingly studied, the broader systemic influences of neuroimmune signaling in PDAC remain poorly understood. Systemic factors, such as hormonal feedback loops and central nervous system modulation, may play a significant role in shaping neuroimmune responses and tumor progression. For instance, the hypothalamic–pituitary–adrenal axis, which regulates stress responses through the release of cortisol and other hormones, has been implicated in modulating immune function and tumor growth in other cancers. Similarly, the central nervous system can influence peripheral immune responses through autonomic nervous system signaling, which may impact PDAC progression.

Emerging evidence suggests that psychosocial stress, which activates both the sympathetic nervous system and the hypothalamic–pituitary–adrenal axis, can promote tumor growth by creating an immunosuppressive microenvironment. In PDAC, stress-induced release of catecholamines and glucocorticoids may suppress anti-tumor immune responses and enhance tumor cell survival. However, direct evidence linking systemic neuroimmune signaling to PDAC progression is currently limited, highlighting a critical gap in the field. Future studies should aim to elucidate how systemic neuroimmune signaling, including hormonal feedback loops and central nervous system modulation, influences PDAC biology and whether targeting these pathways could offer novel therapeutic opportunities.

## 5. Neuroimmune Crosstalk in PDAC

Neuroimmune crosstalk, which can serve as a crucial immunomodulatory hub, has been reported in both healthy and disease states [8,26,27,28]. Accumulating data support the concept that neural circuits, including somatosensory and autonomic divisions, mediate host defense strategies through direct influence on immune regulation and inflammatory pathways in peripheral systems [29,30]. For instance, enteric neurons can reduce surgery-induced intestinal inflammation and prevent postoperative ileus through regulation of mucosal macrophages [31]. Nerve fibers of mouse lymph nodes have close contacts or associations with immune cells, including dendritic cells, macrophages, and T or B lymphocytes [17]. Furthermore, neural regulation of immunological processes through the release of cytokines and neurotrophic factors significantly modulates the proliferation, differentiation, and migratory patterns of endogenous neural stem cells [29,30,32]. For example, enteric neurons maintain the development of muscularis macrophages in a steady state by secreting colony stimulatory factor 1; reciprocally, muscularis macrophages can activate intestinal neurons expressing bone morphogenetic protein receptors by secreting bone morphogenetic protein 2 [33].

Neuroimmune regulation is related to neoplastic diseases and plays a specific role in the occurrence and development of tumors. In a mouse model of triple-negative breast cancer, sciatic nerve stimulation can inhibit tumor growth by activating natural killer cells in the tumor microenvironment [34]. The sympathetic nervous system can maintain the inflammatory microenvironment and promote the occurrence of hepatocellular carcinoma by activating the a1-adrenergic receptors of Kupffer cells [35].

PDAC is a highly aggressive tumor, with a five-year survival rate of only approximately 10% [36]. Pathological and immunohistochemical examinations of PDAC tissue have revealed specific nervous and immune system features. The tumor microenvironment is closely related to neoplastic development [37,38,39,40,41], and neural signals play a role in the tumor microenvironment [42]. Neurotropism is an important histological feature of PDAC that is associated with frequent neurological invasion [43]. In addition, perineural invasion is a common feature of PDAC and is associated with impaired immune responses [11].

**Figure 1:**
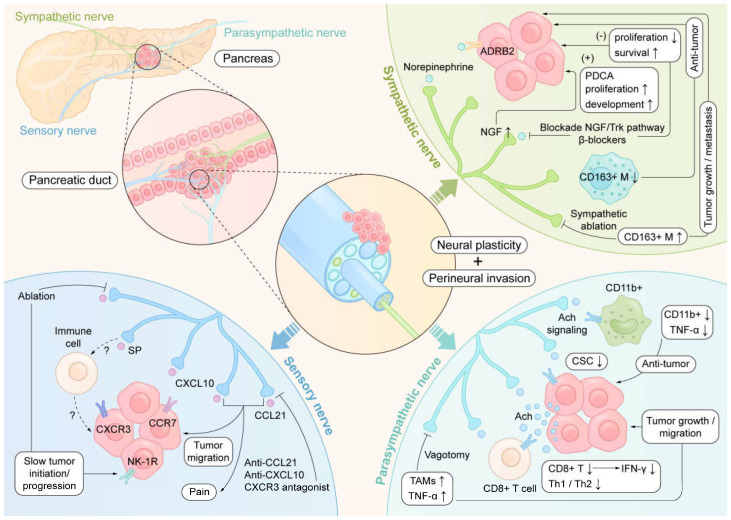
The schematic illustrates the complex interplay between sympathetic nerves, parasympathetic nerves, sensory nerves, and immune cells within the tumor microenvironment of pancreatic ductal adenocarcinoma (PDAC). Key interactions include: (1) Sympathetic nerve signaling via adrenoceptor beta 2 (ADRB2) and its role in tumor progression [10,47]; (2) Parasympathetic nerve signaling via acetylcholine (Ach) and its impact on cancer stem cell (CSC) activity [12,48]; (3) Sensory nerve involvement through substance P (SP) and neurokinin-1 receptor (NK-1R) pathways, which promote tumor migration and immune modulation [25]. Additionally, the figure highlights potential therapeutic interventions, such as CXCR3 antagonists and anti-CCL21 strategies, targeting neuroimmune crosstalk in PDAC [49]. Ach, acetylcholine; IFN-γ, interferon-γ; TAMs, tumor-associated macrophages; TNF-α, tumor necrosis factor-α; NGF, nerve growth factor.

In pancreatic cancer, nerves are significantly infiltrated by cytotoxic T lymphocytes, macrophages, and mast cells, which account for 35%, 39%, and 21%, respectively, of all perineural inflammatory cells [5]. Perineural invasion can induce neuronal damage and inflammation [44]. Colocalization of nerve and immune cells may affect nerve and immune functions, and this neuroimmune regulation may lead to changes in disease. Nerves can regulate tumor growth in ways that affect tumor metabolism. For example, nerves promote PDAC growth in nutrient-deficient environments by releasing substances necessary for tumor growth, such as serine [45]. The neuroimmune interactions occurring in PDAC at the peripheral nerve level, including the regulation of immune cells and sympathetic, parasympathetic, and sensory nerves, are summarized in Figure 1. Despite the promising achievements of tumor immunotherapy in many tumor types, it has not been very successfully applied in PDAC treatment [46]. Determining the mechanisms of neuroimmune regulation in pancreatic cancer may facilitate the progress of tumor immunotherapy for pancreatic cancer treatment.

### 5.1. Sympathetic Nerves

Different interactions between a variety of nerves and immune cells in the pancreas may produce different effects. In prostate cancer, breast cancer, and melanoma, sympathetic nervous system activation has been associated with tumor promotion, primarily through β-adrenoceptor signaling [1]. In the context of PDAC, sympathetic nervous system signaling has been implicated in tumor growth and progression. Studies have shown that β-adrenoceptor signaling contributes to PDAC development at all stages, including precancerous conditions [47,50]. However, the mechanisms underlying these effects are multifaceted and involve interactions with immune cells and other components of the tumor microenvironment. Recent research in mouse models of PDAC has provided insights into the dual role of sympathetic nerves in tumor progression. While sympathetic signaling has traditionally been associated with tumor-promoting effects, some studies suggest that sympathetic axons may exert protective effects by modulating local immune responses [10]. For example, sympathetic nerves have been shown to slow PDAC progression by inhibiting CD163^+^ macrophage subsets at the lesion site. Ablation of sympathetic nerves, on the other hand, leads to increased tumor growth and spread, suggesting that CD163^+^ macrophages may mediate the pro-tumor effects of sympathetic nerve resection [10]. These findings highlight the intricate balance between sympathetic nerve activity and immune cell function in the tumor microenvironment. In melanoma-bearing mice, beta-adrenergic receptor blockers increase the frequency of effector CD8^+^ T cells in tumors and reduce expression of programmed death receptor-1 [51]. While these findings provide valuable insights into the potential therapeutic targeting of sympathetic signaling, it is important to note that the effects of sympathetic nerves on tumor behavior are likely influenced by a variety of factors, including the specific cancer type, stage of disease, and immune contexture. Further research is needed to fully elucidate the mechanisms by which sympathetic nerves influence PDAC progression and to determine whether these findings can be translated to human patients.

### 5.2. Parasympathetic Nerves

The neural effects of parasympathetic nerves are primarily mediated through muscarinic receptors, which play a complex role in modulating immune responses and tumor behavior [1]. Emerging evidence suggests that parasympathetic nervous system input may inhibit primary and secondary tumorigenesis in PDAC through cholinergic signaling. For instance, stimulation of muscarinic type 1 receptors in murine PDAC models has been shown to suppress the cancer stem cell compartment, reduce CD11b^+^ myeloid cell infiltration, lower tumor necrosis factor α (TNF-α) levels, and inhibit metastatic growth in the liver [12]. Conversely, vagotomy, which disrupts parasympathetic signaling, has been associated with increased tumor-associated macrophage (TAM) infiltration, elevated TNF-α levels, and accelerated tumor growth, ultimately reducing survival in a murine pancreatic cancer model [48]. These findings suggest that parasympathetic signaling may exert anti-tumor effects by modulating immune cell activity and inflammatory responses within the tumor microenvironment. However, the precise mechanisms underlying these effects remain to be fully elucidated, and further research is needed to determine whether these observations can be translated to human PDAC.

### 5.3. Sensory Nerves

Sensory nerves also play a significant role in PDAC progression, although their interactions with immune cells and tumor behavior are complex and context-dependent. In genetically engineered PDAC mouse models, sensory innervation increases during the formation of pancreatic intraepithelial neoplasia and further intensifies as the cancer progresses [25]. Sensory neurons secrete chemokines, such as CCL21 and CXCL10, which promote PDAC metastasis by binding to their respective receptors, CCR7 and CXCR3, on tumor cells [49]. Ablation of sensory neurons in these models delays pancreatic intraepithelial neoplasia formation and prolongs survival, suggesting that sensory nerves may facilitate early tumorigenesis and progression [44]. Additionally, Schwann cells, which are associated with peripheral nerves, are activated during the preneoplastic stage and exhibit a strong affinity for cancer cells, initiating nerve–cancer cell interactions [52,53]. For example, TIMP1 secreted by pancreatic cancer cells stimulates Schwann cells and promotes peripheral nerve invasion, while TIMP1 knockdown suppresses this invasive behavior [54]. These findings highlight the multifaceted role of sensory nerves and associated cells in PDAC progression, although the exact mechanisms and their relevance to human disease require further investigation.

Major depressive disorder is four times more common in cancer patients than in the general population [55]. Psychosocial stress activates the sympathetic nervous system, hypothalamic–pituitary–adrenal axis, and neuroendocrine axis and subsequently regulates inflammatory responses through immune cells [56]. In cancer patients, psychosocial stress promotes inflammation and oxidative stress, decreased immunosurveillance, and dysfunctional activation of the autonomic nervous system and the hypothalamic-pituitary-adrenal axis [55]. Human and animal studies have shown that sympathetic and neuroendocrine responses to psychosocial stress significantly influence cancer by regulating inflammatory mediators [56]. For instance, psychological interventions in regional breast cancer patients enhance natural killer cell cytotoxicity and T-cell proliferation and reduce the risk of death after recurrence [57].

## 6. Neuroimmune Regulation Intervention

With advances in surgical techniques, surgical treatment can be used for an increasing number of patients with PDAC [58]. In addition, surgical denervation has long been reported for pain management in patients with PDAC who cannot undergo surgical tumor removal [59]. To date, many intervention studies related to neuroimmune interactions have been conducted, and some of the intervention methods related to PDAC are summarized in Figure 1. In clinical practice, beta-adrenergic receptor blockers, such as propranolol, are the most commonly used drugs to attenuate sympathetic adrenergic function. Patients with PDAC who were administered beta-adrenergic receptor blockers had a lower cancer-specific mortality rate than did those not given these drugs [60]. Compared with conventional receptor blocker drugs, use of a liposome nanomedicine system to administer propranolol hydrochloride can avoid the adverse effects caused by inhibition of nontargeted adrenergic neural activity [61]. Furthermore, it has considerable therapeutic effects on PDAC, prostate cancer, and melanoma, with increased safety and efficiency [61].

There are very precise optogenetic and pharmacogenetic methods that allow neurons to be manipulated in very specific ways [62]. These methods may be developed for the pancreas in the future to achieve desired results. In general, pancreatic neuroimmune regulation will develop in the direction of less trauma, strong targeting, and convenient use.

In addition to the use of systemic drugs, there are many methods for direct neurological intervention, including neurotomy, nerve ablation, and nerve blocks. Perineural invasion is associated with an immunosuppressive microenvironment in PDAC, and intervention involving perineural invasion with bilateral subdiaphragmatic vagotomy is associated with increased CD8^+^ T cells, an increased Th1/Th2 ratio, and improved survival [11]. In contrast to surgery, ultrasound-guided celiac plexus blocks and neurolysis are used to relieve and treat pain in patients with PDAC [63].

In the past few decades, electrical stimulation therapy has attracted considerable attention as a new therapeutic method. The electric field enhances keratinocyte migration, strengthens immune defense, and improves mitochondrial function, and a weak electric field can be safe and has antibacterial effects on the human body [64]. Compared with traditional drugs, which require systematic administration and a certain amount of time for effect depending on the administration, electrical stimulation can act directly on nerve bundles to produce rapid effects [65]. As a bioelectric therapy, stimulation of the vagus nerve in epilepsy patients with an implanted device reduced levels of TNF, interleukin-1β, and interleukin-6 in peripheral blood [66]. More recently, electrical stimulation of the pancreatic nerve was shown to prevent autoimmune diabetes by inhibiting the migration of diabetogenic T cells from pancreatic draining lymph nodes to islets, resulting in less severe insulitis [9].

In addition to confirming the research value, some experimental teams have obtained inconsistent results after repeating the above experiment [67]. Moreover, it should be kept in mind that stimulation of the vagus nerve involves afferent and efferent nerves and controls multiple tissues and organs; in fact, stimulation of the vagus nerve not only causes the desired therapeutic effects but also nerve stimulus-related side effects [9]. Therefore, enhancing the targeting of electrical stimulation therapy to reduce side effects can improve the clinical application prospects of electrical stimulation. However, there are still many problems to be solved, such as the design of the electrode to stimulate the pancreatic nerve, the setting of electrical stimulation parameters, and the method of electrode implantation.

## 7. Concluding Remarks and Future Perspectives

Neuroimmune crosstalk in PDAC offers therapeutic potential through beta-adrenergic blockade, nerve stimulation, and neuropeptide inhibition. However, preclinical successes face clinical translation barriers, such as non-specific interventions disrupting systemic neural balance (e.g., beta-blocker side effects) and patient-specific variability in neural-immune-genetic profiles. For instance, adrenergic inhibition shows inconsistent clinical efficacy due to molecular subtype heterogeneity. Emerging tools like single-cell/spatial omics resolve neuroimmune interactions at unprecedented resolution, identifying Schwann–immune clusters driving immune evasion and neuron–immune synaptic hubs. Optogenetics enables precise neuronal subpopulation manipulation (e.g., targeting protumoral TRPV1^+^ sensory neurons) to refine therapeutic specificity. Clinically, biomarker-driven trials stratifying patients by neural infiltration or neuropeptide receptor expression could enhance response rates. Combining neuroimmune modulation with immunotherapy or stromal reprogramming may overcome resistance, though long-term safety risks (e.g., metastasis promotion) require scrutiny. In summary, realizing neuroimmune therapies demands precision approaches which address mechanistic complexity and patient heterogeneity. Integrating next-gen tools with clinically informed models will unlock targeted PDAC treatment strategies.

Neuroimmune interactions are emerging as key therapeutic targets in PDAC, yet critical gaps persist. First, mechanisms driving dynamic neuroimmune crosstalk—especially during metastatic transition—remain unclear. Second, clinical translation is hindered by unproven long-term efficacy/safety of interventions like neural ablation or neurotransmitter blockade. Key priorities include spatiotemporal mapping, patient-specific targeting, nerve-stromal reprogramming, and so on. Artificial intelligence-enhanced technologies must bridge mechanistic discovery and precision clinical translation, addressing patient variability and long-term outcomes to realize neuroimmune-targeted PDAC treatments.

## Data Availability

Not applicable.

## Abbreviations

The following abbreviations are used in this manuscript:

PDAC	Pancreatic ductal adenocarcinoma
Ach	Acetylcholine
ADRB2	Adrenoceptor beta 2
CSC	Cancer stem cell
IFN-γ	Interferon-γ
SP	Substance P
TAMs	Tumor-associated macrophages
TNF-α	Tumor necrosis factor-α
NGF	Nerve growth factor
NK-1R	Neurokinin-1 receptor
